# The macrophage low-grade inflammation marker sCD163 is modulated by exogenous sex steroids

**DOI:** 10.1530/EC-13-0067

**Published:** 2013-11-18

**Authors:** Henrik H Thomsen, Holger J Møller, Christian Trolle, Kristian A Groth, Anne Skakkebæk, Anders Bojesen, Christian Høst, Claus H Gravholt

**Affiliations:** 1 Medical Research Laboratories, Department of Endocrinology and Internal MedicineClinical Institute, Aarhus University HospitalNørrebrogade 44DK-8000, Aarhus CDenmark; 2 Department of Clinical BiochemistryAarhus University HospitalAarhusDenmark; 3 Department of Molecular MedicineAarhus University HospitalAarhusDenmark; 4 Department of Clinical GeneticsVejle Hospital, Sygehus LillebaeltVejleDenmark

**Keywords:** inflammation, sCD163, Turner syndrome, sex steroids, Klinefelter syndrome

## Abstract

Soluble CD163 (sCD163) is a novel marker linked to states of low-grade inflammation such as diabetes, obesity, liver disease, and atherosclerosis, all prevalent in subjects with Turner syndrome (TS) and Klinefelter syndrome (KS). We aimed to assess the levels of sCD163 and the regulation of sCD163 in regards to treatment with sex hormone therapy in males with and without KS and females with and without TS. Males with KS (*n*=70) and age-matched controls (*n*=71) participating in a cross-sectional study and 12 healthy males from an experimental hypogonadism study. Females with TS (*n*=8) and healthy age-matched controls (*n*=8) participating in a randomized crossover trial. The intervention comprised of treatment with sex steroids. Males with KS had higher levels of sCD163 compared with controls (1.75 (0.47–6.90) and 1.36 (0.77–3.11) respectively, *P*<0.001) and the levels correlated to plasma testosterone (*r*=−0.31, *P*<0.01), BMI (*r*=0.42, *P*<0.001), and homeostasis model of assessment insulin resistance (*r*=0.46, *P*<0.001). Treatment with testosterone did not significantly lower sCD163. Females with TS not receiving hormone replacement therapy (HRT) had higher levels of sCD163 than those of their age-matched healthy controls (1.38±0.44 vs 0.91±0.40, *P*=0.04). HRT and oral contraceptive therapy decreased sCD163 in TS by 22% (1.07±0.30) and in controls by 39% (0.55±0.36), with significance in both groups (*P*=0.01 and *P*=0.04). We conclude that levels of sCD163 correlate with endogenous testosterone in KS and are higher in KS subjects compared with controls, but treatment did not significantly lower levels. Both endogenous and exogenous estradiol in TS was associated with lower levels of sCD163.

## Introduction

The CD163 molecule, expressed by cells of the monocyte lineage, particularly the macrophages, is part of a scavenger system with a high affinity for the hemoglobin–haptoglobin complex. It contains nine scavenger-receptor cysteine-rich domains that are located on the extracellular side of the cell membrane (1). CD163 is expressed at different levels in different organs and in response to varying local chemical signals (2). One of its main and well-described functions is the removal of plasma hemoglobin through endocytosis of the very high-affinity complex hemoglobin–haptoglobin, thus preventing the oxidative stress from free hemoglobin by the release of the free iron, bilirubin, and carbon monoxide. Glucocorticoids and anti-inflammatory cytokines such as IL6 and 10 induce increased CD163 expression thus assigning CD163 anti-inflammatory effects. Its proinflammatory effects are seen by the downregulation of CD163 by inflammatory cytokines such as tumor necrosis factor α (TNFα) and granulocyte–macrophage colony-stimulating factor (2, 3). A soluble form of CD163 (sCD163) is formed by proteolytic cleavage of the extracellular part of the protein and shed into circulation (1, 2). The function of sCD163 is not clear; however, a role in the elimination of *Staphylococcus aureus* has recently been described (4) in addition to the findings of anti-inflammatory effects through inhibition on T lymphocyte activation and proliferation (2, 3).

Recently increased plasma levels of sCD163 have been linked to states of low-grade inflammation such as diabetes, obesity, liver disease, and atherosclerosis (5, 6, 7, 8, 9, 10, 11), underscoring the important role of macrophages in initiating and propagating these conditions. Previously, we and others have shown that both females with Turner syndrome (TS) and males with Klinefelter syndrome (KS) show evidence of low-grade inflammation (12, 13, 14) and this substantiates the increased frequency of type 2 diabetes, disease of the circulatory system including valvular heart disease, pulmonary embolism, but excluding ischemic heart disease in KS, and aortic valve disease, hypertension, aortic aneurysm, and ischemic heart disease in TS (15, 16, 17, 18, 19, 20).

The changes seen in TS and KS is not fully understood and it is not known if the changes are entirely due to hypogonadism and most patients will require treatment with sex hormones for long periods of their lives. The low-grade inflammation seen in KS and TS are influenced by the low levels of sex hormones (12, 13), 17β-estradiol (E_2_) and testosterone, that are known to modulate the inflammatory state of macrophages (21, 22). An experiment of testosterone and E_2_ cultured Hofbauer cells (fetal macrophages) found no influence on CD163 expression (23).

The aim of the present study was firstly to investigate the regulation of the sCD163 in TS and KS and its relation to other markers of low-grade inflammation and secondly to investigate whether treatment with sex hormones, i.e. estrogen–progestin and testosterone, would impact circulating levels of sCD163.

To that end we studied samples drawn from one study with KS, one with TS (24, 25, 26), and one experimental study of acute male hypogonadism. We hypothesized that hypogonadism in both sexes would be accompanied by raised levels of sCD163 and that this is associated with other markers of low-grade inflammation.

## Subjects and methods

### KS cross-sectional study

Seventy subjects with KS were recruited from fertility and endocrine outpatient clinics and compared with healthy age-matched controls (*n*=71) recruited from at the University of Aarhus and the Blood Bank of Aarhus University Hospital. Inclusion and exclusion criteria were as previously described (26). Half of the KS were receiving testosterone supplementation with testosterone injections (*n*=20), testosterone undecanoate (*n*=14), and mesterolon (*n*=1), whereas the other half did not receive treatment. All received oral and written information concerning the study before giving written informed consent. Data regarding glucose and bone mineral metabolism have been presented previously (12, 24). The protocol was approved by the Aarhus County Ethical Scientific Committee (no. 20010155) and the Danish Data Protection Agency.

### Experimental male hypogonadism study

Twelve healthy, nonsmoking male volunteers participated in this study. All volunteers displayed normal primary and secondary sex characteristics and none of them used medication or had a positive family history of diabetes. Men who were planning to participate in competitive sport events during the subsequent year were not included. All had levels of testosterone 18.6 (8.3–32.9) nmol/l as well as luteinizing hormone (LH) 4.8 (1.7–8.1) IU/l and follicle-stimulating hormone (FSH) 3.2 (1.2–6.6) IU/l within the normal range. Other details on the study group have been described previously (27). In short, hypogonadism was achieved by s.c. injection of gonadotropin-releasing hormone agonist (7.5 mg leuprorelide, Eligard, Astellas Pharma, Wallisellen, Switzerland) before three of four trial sessions. Thus, hypogonadal trial days were preceded by at least 7–10 days of castrate levels of testosterone, designed to achieve stable changes in their metabolic state. The four study arms were three hypogonadal arms with either a 50- or 150-mg of testosterone gel or a placebo gel applied along with an eugonadal control arm. Trial sessions included baseline measurements and testosterone treatment. Insulin sensitivity was assessed using the hyperinsulinemic and euglycemic clamp technique. All volunteers received oral and written information concerning the study before giving written and informed consent. The protocol was approved by the Aarhus County Ethical Scientific Committee (no. M-20070046), registered at ClinicalTrials.gov (NCT-00613288), and performed in accordance with the Helsinki Declaration II.

### TS treatment study

A total of eight subjects with TS were compared with eight age-matched healthy controls. The design was a randomized crossover study. Both groups underwent a 2 month wash-out period from hormone replacement therapy (HRT) and oral contraceptive therapy (OCT) respectively. Subjects were examined at the end of each 2-month period. The treatment consisted of 2 mg E_2_/day (days 1–22), 1 mg norethisterone/day (days 13–22), and 1 mg E_2_/day (days 23–28) (Trisekvens, Novo Nordisk A/S, Copenhagen, Denmark) for the subjects with TS. The controls received combined contraceptive pills. Other details on the study group have been described previously (26, 28). All subjects received oral and written information concerning the study before giving written informed consent. The protocol was approved by the Aarhus County Ethical Scientific Committee (no. 1996/3561).

### Assays

The plasma concentration of sCD163 was determined in duplicate in samples that had been frozen at −20 °C by an in-house sandwich ELISA using a BEP-2000 ELISA-analyzer (Dade Behring, Deerfield, IL, USA) essentially as previously described (25). The duration of storage was 2–8 years. Briefly, rabbit anti-CD163 (2 mg/l) was coated onto microtiter wells and plates were transferred to a BEP-2000 ELISA-analyzer (Dade Behring, Eschborn, Germany). Samples (diluted 1:101) were added in duplicates and incubated for 1.5 h at 37 °C. Monoclonal anti-CD163 (GHI/61, 3 μg/ml) was then added for 1 h at 37 °C, followed by incubation for 1 h at 37 °C with HRP-labeled goat antimouse antibodies (0.125 μg/ml; Dako, Glostrup, Denmark). The plates were developed with tetramethylbenzidine substrate solution (Kem-En-Tec, Taastrup, Denmark). The assay was calibrated using serum traceable to purified human CD163, with the lowest calibrator being 6.25 μg/l. The inter-assay coefficient of variation on control samples included on each plate (15 runs) was 3.6% at 1.90 mg/l and 4.4% at 3.61 mg/l.

Adiponectin was determined by use of an in-house time-resolved immonoflourometric assay (TR-IFMA) as described (29). Leptin was determined by a commercial RIA (Linco, St Louis, MO, USA). Total insulin-like growth factor 1 (IGF1) was measured by use of in-house noncompetitive, TR-IFMAs after acid–ethanol extraction of serum as described (30). C-reactive protein (CRP) was measured by an ultrasensitive assay (Diagnostic Products, Los Angeles, CA, USA). Androgens, estrogens, sex hormone-binding globulin, LH, and FSH were analyzed as described (24).

### Statistical analysis

We used paired samples *t*-test and otherwise independent samples *t*-test, as appropriate. Correspondingly, Mann–Whitney U test or Wilcoxon's signed-rank test was used in the analysis of nonparametric data. We used Pearson's or Spearman's coefficient of correlation as appropriate. In the KS cross-sectional study, we compared untreated males with KS and controls, and then untreated vs testosterone-treated males with KS. Because sCD163 was correlated to a host of variables, we performed stepwise multivariate regression analysis in order to evaluate the impact of independent variables on the dependent variable, sCD163, in the KS group and healthy subject group separately and combined. Significance level for entering and for removal of variables from the model was *P*<0.05 and *P*>0.10 respectively. We had no valid information on timing of the last i.m. injection of testosterone among KS. Likewise, untreated females with TS were compared with untreated controls and then untreated and treated females with TS were compared. All results are shown as mean±s.d. or median±range as appropriate. Statistical analysis of data was carried out using the SPSS Software (SPSS, Inc., Chicago, IL, USA), version 20 for Windows. For the experimental hypogonadism study, statistical comparisons for groups over time were analyzed by repeated-measures ANOVA. One-way ANOVA was used to analyze base line data (*t*=−120) and at the end of the clamp period (*t*=360). We considered three statistical models of relevance: the full ‘4’ arm model, the ‘3’ arm model consisting of the hypogonadal arms only, thereby assessing the acute intervention with the placebo arm as the functional ‘control’ arm, and lastly the ‘2’ arm model comparing sustained hypogonadism with the eugonadal state.

## Results

### KS cross-sectional study

**Figure 1:**
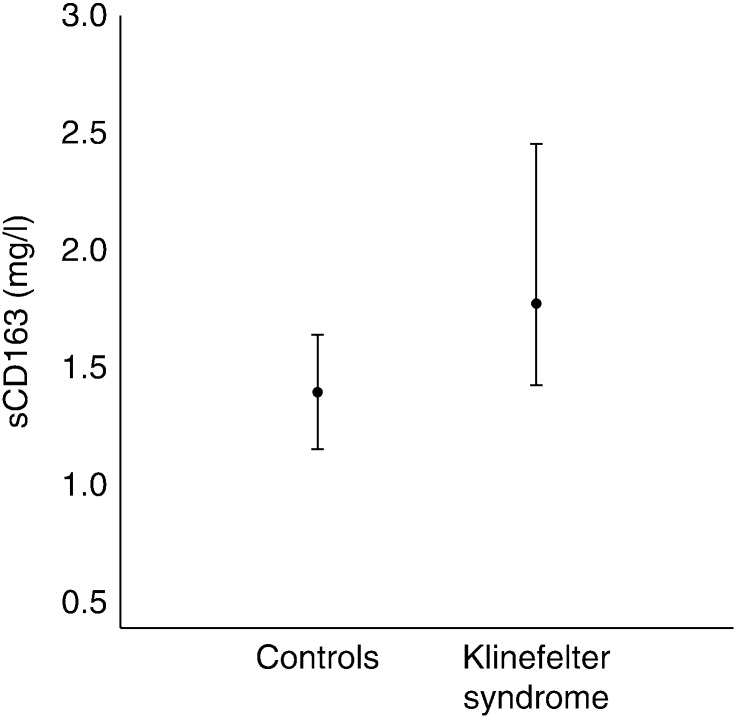
Klinefelter syndrome cross-sectional study. Data are presented as median with 25th and 75th percentiles. *P* value <0.001.

**Figure 2:**
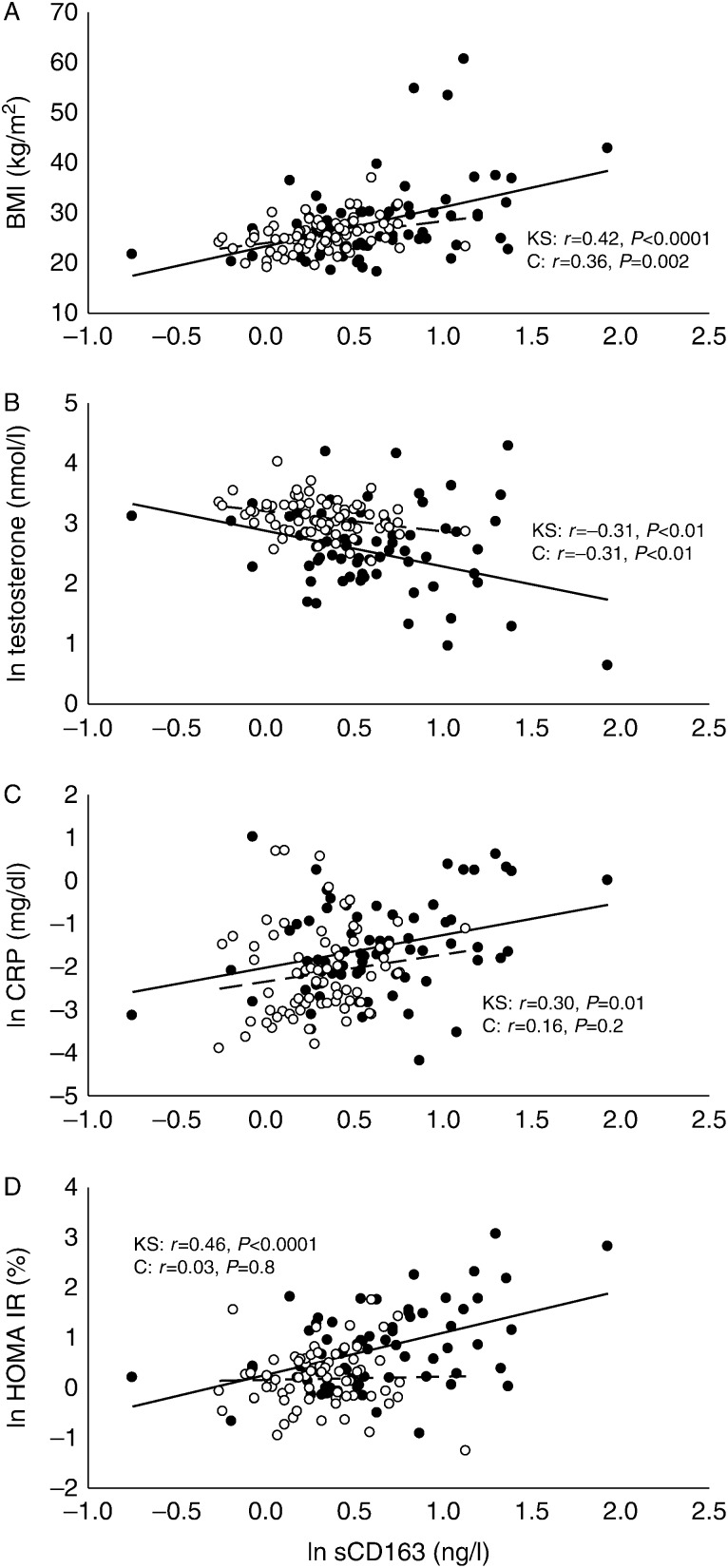
Data obtained from Klinefelter syndrome cross-sectional study represented as regression line and scattered graph plotted for sCD163 against different variables. sCD163 plotted against different variables. Open circles represent controls and filled circles represents KS subjects. Regression lines are inserted: solid lines indicate KS and broken lines indicate controls. *P* and *r* values are inserted in all figures. (A) BMI. sCD163 is positively and uniformly associated with BMI in both the KS and control group. (B) Testosterone correlated with sCD163 in the two groups. (C) CRP correlated with sCD163 in both groups. (D) Insulin sensitivity (HOMA IR) correlated with sCD163 in KS subject, but not in controls.

Characteristics of KS and controls are presented in Table 1. As previously described, the control subjects were significantly leaner than the subjects with KS, had a lower body fat (BF) percentage, and a higher lean body mass (LBM). KS had a significantly higher prevalence of diabetes and their metabolic profile was altogether less favorable with regards to insulin resistance, obesity, diabetes, and hypertension (12). The level of sCD163 was significantly higher in the KS group by 29% (Fig. 1). There was no significant difference in levels between untreated and treated KS subjects (2.00 (0.83–4.03) vs 1.72 (0.47–6.90) mg/l, *P*=0.18). We therefore analysed the correlation between sCD163 and other variables in the whole KS group (treated and untreated). In KS subjects, sCD163 correlated with BMI (*r*=0.360, *P*<0.001), LBM (*r*=−0.310, *P*=0.02), and other body measures such as total BF, fat on trunk, waist:hip ratio, or waist circumference. Furthermore, there was correlation with testosterone (*r*=−0.306, *P*<0.01) and other androgens, homeostasis model of assessment insulin resistance (HOMA IR; *r*=0.456, *P*<0.001), VO_2_ max (*r*=−0.262, *P*=0.04), IGF1 (*r*=−0.297, *P*=0.01), and CRP (*r*=0.302, *P*=0.01). In controls sCD163 correlated with BMI (*r*=0.360, *P*=0.002), LBM (*r*=−0.283, *P*=0.02), and similar body measures. Among controls, the testosterone (*r*=−0.306, *P*=0.01) also correlated with sCD163 along with VO_2_ max (*r*=−0.27, *P*=0.02), but not with HOMA IR, CRP, dehydrotestosterone, and IGF1 (Table 2 and Fig. 2). Subsequent multiple linear regression analyses in the combined population of KS and controls was done with sCD163 as the dependent variable. BMI, testosterone, and status (KS or control) were the only independent variables (*r*=0.579, *P*<0.0001). Thus, these variables explain about 35% of the variation in sCD163, with prominent differences between KS and controls.

### Experimental hypogonadism study

To investigate the direct effect of testosterone on sCD163 levels, we included data from an experimental hypogonadism study on healthy male volunteers. Baseline characteristics and levels of testosterone have been presented before (27). As expected, no differences in parameters reflecting body composition were seen during short-term hypogonadism. Likewise, the concentrations of triglycerides (TGs), VLDL–TG, free-fatty acids, cortisol, insulin, glucose, and glucose infusion rates were comparable during both basal and clamp periods in all statistical models (data not shown). Short-term hypogonadism did not affect the levels of sCD163 (*P*=NS, basal period, one-way ANOVA), nor did testosterone treatment in any model affect the levels at the end of a 3 h hyperinsulinemic euglycemic clamp (*P*=NS, one-way ANOVA, and repeated measures ANOVA respectively).

### TS treatment study

**Figure 3:**
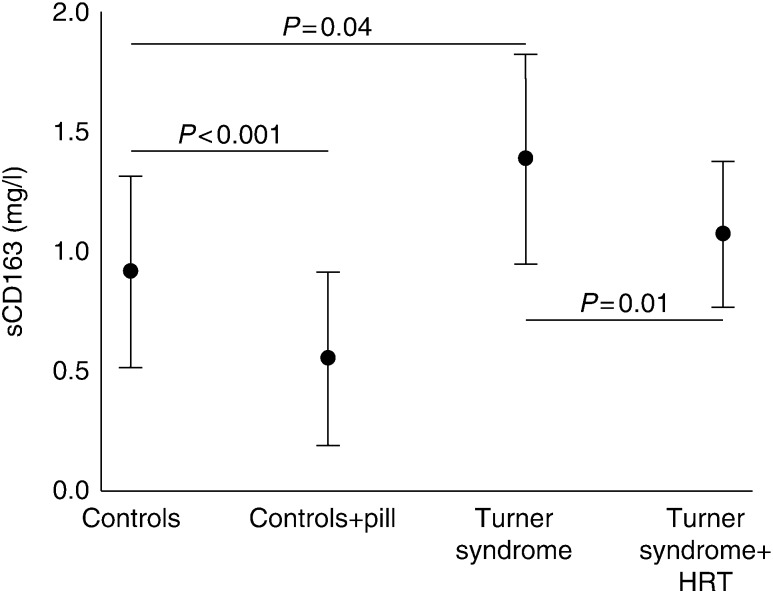
Turner syndrome treatment study. Data are presented as mean±s.d. *P* values are indicated in the figure.

Baseline characteristics for TS and controls are presented in Table 3. As previously described, controls and TS had similar BMI, fat mass, and fat-free mass as well as similar HOMA IR. TS had lower levels of E_2_ and testosterone and higher levels of FSH and LH (31). In the untreated state, sCD163 was significantly higher among TS subjects compared with controls (Fig. 3). Treatment with HRT or contraceptive pills (with ethinyl E_2_ as the active ingredient) respectively significantly lowered levels of sCD163 in both TS and controls (Fig. 3). Our E_2_ assay does not pick up ethinyl estradiol, hence suppressed levels of E_2_ is seen in the controls receiving OCT. CRP was significantly higher in the TS subjects in the untreated situation compared with control group. During active treatment, CRP increased in controls due to OCT, while it did not change significantly in TS subjects. There was no correlation between sCD163 and any of the other measured variables in either TS or controls.

## Discussion

This study shows that sCD163, as a macrophage-based marker of chronic low-grade inflammation, is elevated in both TS and KS. In addition, we show that sCD163 is influenced by both endogenous testosterone, and endogenous and exogenous E_2_ and norethisterone, which is a novel finding.

Whether presence of low-grade inflammation has any clinical significance in the disorders of chromosomal anomalies is yet to be investigated, but we know from previous studies that the risk of type 2 diabetes and cardiovascular diseases are markedly increased in both TS and KS (19, 20, 32, 33). Markers of low-grade inflammation have been shown to predict mortality and morbidity in various diseases (6, 34, 35, 36, 37, 38). Males with KS suffer from low-grade inflammation compared with age- and sex -matched controls, as shown here by elevated sCD163, but also shown previously with other markers of low-grade inflammation like CRP (12, 26). We saw no difference in the levels of sCD163 between KS treated with testosterone and untreated KS. In multiple linear regression analyses, we could show that BMI, but also status (i.e. KS or control), remained as independent contributors of the level of sCD163 in the combined study group. We and others have previously shown that abdominal adiposity, insulin resistance, and outright type 2 diabetes are frequent occurrences in KS (12, 33, 39, 40) and low-grade inflammation in subjects with KS could likely attribute to their metabolic phenotype which in turn, at least in part, is due to their relative hypogonadism. Our data do not indicate that treatment with testosterone reverses the low-grade inflammation, as evident by sCD163, because levels were similar in the treated and untreated KS groups. Levels of sCD163 is higher amongst obese subjects compared with lean subjects and positively correlated to various other unfavorable metabolic features (7) and indeed, though in lesser part, to levels of adipocytokines. The failure to demonstrate significant efficacy in lowering sCD163 levels by testosterone treatment in our KS subjects may be due to the duration of treatment. It also has to be kept in mind that the study was not randomized or otherwise designed to prove efficacy of testosterone treatment, but was merely observational.

Previously, we have shown that high-sensitive CRP and TNFα is higher in TS compared with controls and speculated that this is due to a chronic condition with low-grade inflammation (13, 41, 42). We have now shown that this is accompanied by increased sCD163 (TS treatment study), and a striking downregulating effect on sCD163 of both HRT in TS and contraceptive pills in controls (−22 and −39% respectively). Interestingly, the downregulatory effect on sCD163 of HRT in TS and OCT in controls is contrary to the effects on CRP-levels (increased by OCT in controls and unchanged in TS). This finding in controls is in concert with others (43) and suggests that different estrogens, i.e. natural E_2_ and synthetic ethinyl estradiol, do indeed have differential effects on markers of inflammation. Because controls received oral combination contraceptive pills and though TS subjects were sampled in the ‘follicular phase’, any impact on our result from the added progestins is unknown. E_2_ has in macrophages been shown to act anti-inflammatory and suppress TNFα through the suppression of NF-κB activation (44), but the results are not uniform. In a study on women with hyperinsulinemic androgen excess, OCT lead to an increase in *CD163* gene expression contrary to our findings in a hypogonadal model (45). The *CD163* expression correlated to unfavorable metabolic features (e.g. increase in visceral fat). In the other study arm, metformin and flutamide treatment induced a more metabolically favorable profile and lead to a lowering of *CD163* gene expression. This has also been established *in vitro*, where metformin was also shown to downregulate CD163 (46). The reason for the discordant results is probably explained by the distinct metabolic differences between TS and women with PCOS. In a study using isolated human-monocyte-derived macrophages, E_2_ did not affect the production of TNFα or other cytokines, and CRP was affected variably depending on the pertinent level of LDL-cholesterol, with high levels of LDL leading to a larger production of CRP, while low levels of LDL led to diminished CRP (47). Moreover, sCD163 is specifically produced by macrophages, whereas CRP is predominantly produced by hepatocytes. Our results may therefore be interpreted as a specific effect of estrogens on macrophage activity, which does influence, e.g. IL6-mediated hepatic CRP expression. Whether this effect is mediated through changes in metabolic features like BMI and insulin resistance associated with low-grade inflammation themselves (48) cannot be established by the current data and TS subjects do indeed have higher BMI, albeit not significantly in our study, than that of controls and this is not normalized by the short-term treatment with HRT. While the short-treatment duration in this study did not result in changes in body composition measures such as BMI, it is entirely possible that the observed effects on sCD163 is mediated through regulation of adipokines by E_2_/progestin. Proinflammatory cytokines upregulate 11β-hydroxysteroid dehydrogenase type 1 (11β-HSD1) in adipose tissue and higher levels have indeed been associated also with the metabolic syndrome (49) found prevalent in both KS and TS (12, 41). Thus at least at a localized level, increased glucocorticoid action might contribute to the higher levels of sCD163 (2) as found in our study via the mechanisms not yet understood. While glucocorticoids are potent inducers of increments in CD163 expression, this does not necessarily increase levels of sCD163 in plasma, but when accompanied by an inflammatory stimulus it gives rise to increased cleavage activity of a disintegrin and metalloproteinase 17 known as ADAM17 and TNFα converting enzyme (TACE), resulting in increased shedding of sCD163 from the cell surface of the glucocorticoid-activated machrophages (50). Neither E_2_ nor testosterone gave rise to increased expression of CD163 in an *in vitro* experiment, pointing out this intangible association between CD163 expression and the soluble form of CD163 (23).

In conclusion, we have shown that the level of sCD163 is influenced by both endogenous and exogenous sex hormones in different states of sex hormone deficiency. As a macrophage-based marker of chronic low-grade inflammation, sCD163 is elevated in both TS and KS and correlates with indices of body composition and markers of insulin resistance. Estrogen–progestin treatment significantly decreases sCD163 in contrast to CRP. The different mechanisms of origin and activation of sCD163 and CRP might prove useful in clinical settings of different etiologies, but warrants further targeted research to establish any firm conclusions. At present, it is not evident whether low-grade inflammation is a result or a consequence of co-existing disease states (26) or may have both protective and harmful effects. Likewise, the association between sex hormones and the immune system needs further research to provide knowledge in a clinical setting.

## Figures and Tables

**Table 1:** Data on KS subjects and controls from the KS cross-sectional study, regarding inflammation markers, body composition, and hormones and insulin sensitivity. Data are mean±s.d. or median (total range).

	**Controls**	**KS**	**U-KS**	**T-KS**	***P* value**		
Controls vs KS^a^	Controls vs U-KS^a^	U-KS vs T-KS^a^
*n*	71	70	35	35			
Age (years)	36.4 (19.2–68.0)	35.5 (19.0–66.2)	35.0 (19.0–66.2)	38.7 (19.3–62.3)			
sCD163 (mg/l)	1.36 (0.77–3.11)	1.75 (0.47–6.90)	2.00 (0.83–4.03)	1.72 (0.47–6.90)	<0.001^b^	<0.001^b^	0.181^b^
Testosterone (nmol/l)	21.8 (10.6–55.5)	12.77 (0.8–72.2)	12.68 (0.8–37.3)	14.04 (1.9–72.2)	<0.001	<0.001	0.192
Estradiol (pmol/l)	81.0 (40–210)	86.0 (40–290)	77 (40–140)	89 (44–290)	0.24	0.819	0.041
BMI (kg/m^2^)	24.9 (19.0–36.9)	26.9 (18.1–60.6)	27.3 (20.0–60.6)	25.1 (18.1–54.7)	0.046	0.008	0.369
LBM (kg)	78.4±6.4	70.4±8.7	68.4±7.3	72.7±9.8	<0.001^c^	<0.001^c^	0.062^c^
BF (%)	18.9±6.8	26.7±9.2	28.7±7.6	24.3±10.4	<0.001^c^	<0.001^c^	0.069^c^
VO_2_ max (ml O_2_/kg per min)	43.5 (24.0–73.3)	29.9 (14.6–57.3)	29.9 (14.6–50.1)	29.9 (14.9–57.3)	<0.001^b^	<0.001^b^	0.943^b^
CRP (mg/l)	0.11 (0.02–1.99)	0.19 (0.02–2.74)	0.21 (0.03–2.74)	0.17 (0.02–1.83)	0.001	0.001	0.103
HOMA IR (%)	1.2 (0.3–5.7)	2.1 (0.04–21.3)	2.3 (0.5–10.0)	1.8 (0.4–21.3)	<0.001^b^	<0.001^b^	0.976^b^
Adiponectin	4.21 (2.3–9.1)	3.7 (1.4–13.6)	3.5 (1.4–9.7)	4.5 (1.6–13.6)	0.53^b^	0.125^b^	0.126^b^
Leptin (ng/l)	3.1 (1–17)	11.0 (2–116)	14.0 (2–116)	8.4 (2–75)	<0.001	<0.001	0.081

KS, Klinefelter syndrome; U-KS, untreated-KS; T-KS, treated-KS; LBM, lean body mass; BF, body fat; VO_2_ max, maximal oxygen uptake; CRP, C-reactive protein; HOMA IR, homeostasis model of assessment insulin resistance.

a Mann–Whitney *U*-rank sum test.

b Student's *t*-test with ln-transformed data.

c Student's *t*-test.

**Table 2:** Data on KS subjects and controls from the KS cross-sectional study, regarding correlation analysis between sCD163 and markers of body composition, hormones, insulin sensitivity, and others.

	**Controls**	***P* value**	**KS**	***P* value**
*n*	71		70	
sCD163 (ln)	1.00		1.00	
Testosterone (ln)	−0.31	<0.01	−0.31	<0.01
BMI	0.36	0.002	0.42	<0.001
Waist:hip ratio	0.33	0.006	0.30	0.01
Fat on trunk	0.21	0.08	0.42	0.001
VO_2_ max	−0.27	0.02	−0.26	0.04
HOMA IR (ln)	0.03	0.82	0.46	<0.001
IGF1 (ln)	−0.19	0.12	−0.30	0.01
Adiponectin (ln)	0.004	0.98	−0.15	0.22
Leptin (ln)	0.29	0.014	0.45	<0.001
Insulin (ln)	0.03	0.82	0.46	<0.001
Cholesterol, total	0.16	0.18	0.08	0.51
Cholesterol, HDL (ln)	−0.10	0.40	−0.25	0.04

Data are Pearson's correlation coefficient (*P* value). KS, Klinefelter syndrome; LBM, lean body mass; BF, body fat; VO_2_ max, maximal oxygen uptake; HOMA IR, homeostasis model of assessment insulin resistance; IGF1, insulin-like growth factor 1.

**Table 3:** Data on TS subjects and controls from the TS treatment study, regarding inflammation markers, BMI, sex hormones, and insulin sensitivity. Data are mean±s.d. or median (total range).

	**Controls**	**TS**	***P* value**	**Controls+OCT**	**TS+HRT**	***P* value**
Controls vs TS^a^	TS vs TS+HRT^b^
*n*	8	8				
Age (years)	28.5±4.2	29.1±5.8				
sCD163 (mg/l)	0.91±0.40	1.38±0.44	0.04	0.55±0.36	1.07±0.30	0.01
BMI (kg/m^2^)	21.9 (20–30)	25.9 (22–31)	0.07^c^	21.7 (19.9–29.9)	25.3 (22.6–33.0)	0.67^d^
Estradiol (pmol/l)	320 (20–610)	110 (90–150)	0.01^c^	115 (70–240)	220 (110–1280)	0.02^d^
Testosterone (nmol/l)	2.18±0.59	1.41±0.77	0.04	1.35±0.41	1.20±0.75	0.32
CRP (mg/l)	0.09 (0.04–0.16)	1.03 (0.15–2.04)	0.01	0.24 (0.12–1.53)	0.73 (0.23–2.50)	0.52^e^
HOMA IR	0.56±0.28	0.71±0.24	0.28	0.60±0.21	0.80±0.43	0.36

TS, Turner syndrome; OCT, oral contraceptive therapy; HRT, hormone replacement therapy; CRP, C-reactive protein; HOMA IR, homeostasis model of assessment insulin resistance.

a Independent samples *t*-test.

b Paired samples *t*-test.

c Mann–Whitney *U*-rank sum test, independent samples.

d Mann–Whitney *U*-rank sum test, dependent samples.

e Paired samples *t*-test with ln-transformed data.
